# Simultaneous Immunofluorescence-Based In Situ mRNA Expression and Protein Detection in Bone Marrow Biopsy Samples

**DOI:** 10.21769/BioProtoc.5612

**Published:** 2026-06-20

**Authors:** Alba Lillo Sierras, Sandro Bräunig, Hongzhe Li, Stefan Scheding

**Affiliations:** 1 Division of Molecular Hematology and Stem Cell Center, Lund University, Lund, Sweden; 2 Department of Hematology, Skåne University Hospital, Lund, Sweden

**Keywords:** Multi-color immunofluorescence staining, Spatial RNA expression analysis, RNA-scope, Bone marrow fibrosis, Acute lymphoblastic leukemia

## Abstract

Fluorescence in situ hybridization (FISH) can be employed to study the expression and subcellular localization of nucleic acids by using labeled antisense strands that hybridize with the target RNA or DNA molecules. Likewise, immunofluorescence antibody staining (IF) takes advantage of the specific interaction between a fluorophore-labeled antibody and its corresponding antigen. This protocol reports the combination of RNA-FISH and IF antibody staining for simultaneous detection of both RNA transcripts and proteins of interest in routine formalin-fixed paraffin-embedded (FFPE) bone marrow biopsy samples. Herein, we provide a detailed description of the methodology that we have developed and optimized to study the spatial expression of two transcripts—TGFB1 and PDGFA1—in human hematopoietic (CD45^+^) and non-hematopoietic (CD271^+^) cells in the bone marrow of patients with acute lymphoblastic leukemia (ALL).

Key features

• The protocol describes the simultaneous visualization of RNA target transcripts and protein expression.

• In situ RNA and surface marker analysis was established for routine human bone marrow biopsies.

## Graphical overview


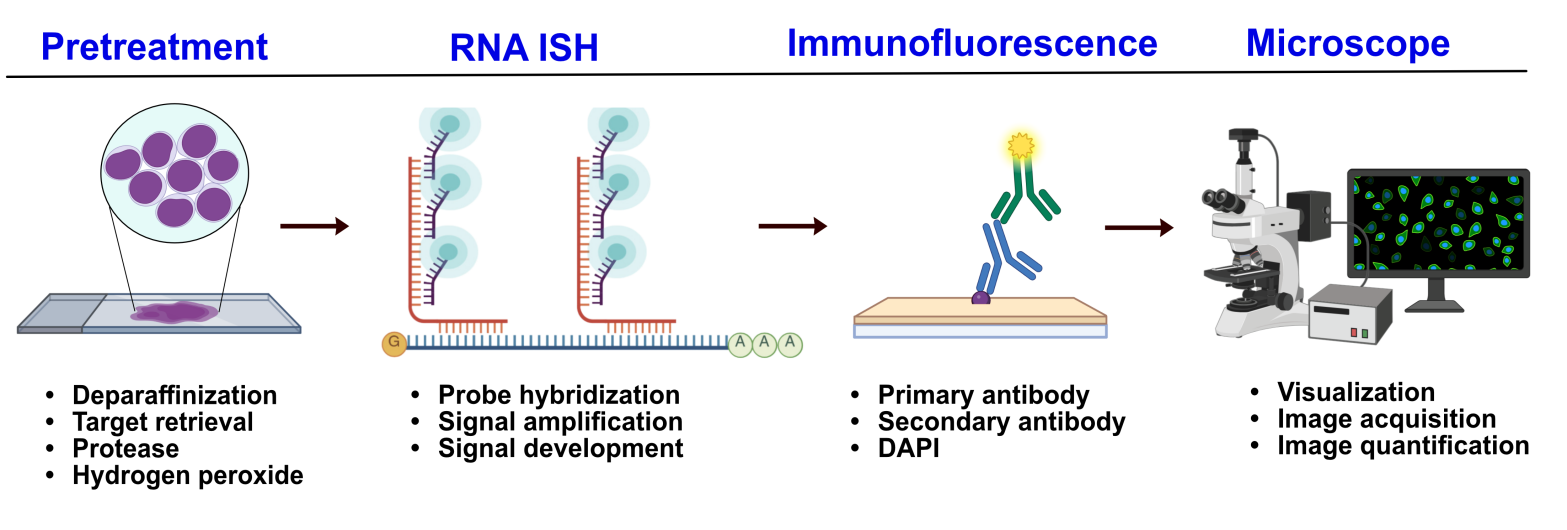


**Dual RNA fluorescence in situ hybridization and protein immunofluorescence workflow**

## Background

Acute lymphoblastic leukemia (ALL) is a blood cancer defined by the aberrant production of lymphoid blast cells. The presence of bone marrow (BM) fibrosis in ALL is correlated with poorer prognosis [1]. However, the mechanisms leading to BM fibrosis in ALL remain unknown. We therefore studied the spatial expression of two cytokines, TGFB1 and PDGFA1, which are known to play a major role in primary myelofibrosis (PMF), in bone marrows of ALL patients in comparison with PMF to assess whether the two diseases share common mechanisms of fibrosis initiation and progression [2].

Real-time RT-PCR is widely used for the detection and quantification of target mRNA, being extremely sensitive. However, the RNA extraction step destroys the tissue, which makes it impossible to spatially assess tissue gene expression. On the other hand, traditional antibody-based fluorescence protein detection preserves tissue architecture but does not provide information about possible transcriptional changes.

In order to simultaneously assess gene and protein expression in bone marrow tissues, we therefore combined the RNAscope^®^ in situ hybridization assay for TGFB1 and PDGFA1 transcripts with antibody-based immunofluorescence (IF) staining using hematopoietic and stromal cell surface markers. The main advantage of using RNAscope^TM^ technology is its high sensitivity and specificity compared to traditional in situ RNA hybridization methods. This is due to the use of double Z probes that allow the detection of lowly expressed or partially degraded RNA while maintaining target-specific signal. Combining the in situ mRNA detection with specific cell marker analysis allowed us to study the expression of our target genes in defined cell types, providing information about cellular heterogeneity and gene expression patterns throughout the tissue. This protocol, however, is currently restricted to the detection of up to three different RNA targets at a time, and the use of additional cell surface markers clearly depends on the spectral compatibility of the fluorophores and the number of channels available on the imaging platform.

## Materials and reagents

**Biological materials**

1. FFPE bone marrow biopsy sections (5 μm thick) from the iliac crest were obtained from the Division of Pathology, Laboratory Medicine Skåne, Lund, Sweden. Sections were routine pathology specimens from nine Philadelphia-negative B-cell ALL (B-ALL) patients (three females and six males, ages ranging from 2 to 7 years old, with fibrosis grades 0–2) and three adult patients with primary myelofibrosis (one male and two females, with ages ranging from 32 to 50 years old). Samples from hematologically normal patients were included as negative controls.

**Reagents**

1. RNAscope^®^ probes

a. RNAscope^®^ probe for *TGFB1* (Hs-TGFB1-no-XMm-C2) (Advanced Cell Diagnostics, catalog number: 443481-C2)

b. RNAscope^®^ probe for *PDGFA1* (Hs-PDGFA-no-XMm-C1) (Advanced Cell Diagnostics, catalog number: 441061)

c. Positive control probe against the human POLA2A PPIB UBC transcripts (Advanced Cell Diagnostics, catalog number: 320861)

d. Negative control probe against the DapB transcript (accession #EF191515) of the *Bacillus subtilis* strain SMY ± TSA Vivid^TM^ Fluorophore 570 and TSA Vivid^TM^ 650 (Advanced Cell Diagnostics, catalog number: 3200871)

2. RNAscope^®^ Multiplex Fluorescent Reagent kit v2 (Advanced Cell Diagnostics, catalog number: 323100)

a. Pretreatment reagents

i. RNAscope^®^ hydrogen peroxide (Advanced Cell Diagnostics, catalog number: 322381)

ii. RNAscope^®^ protease plus (Advanced Cell Diagnostics, catalog number: 322381)

iii. RNAscope^®^ 10× target retrieval (Advanced Cell Diagnostics, catalog number: 322000)

b. RNAscope^®^ Multiplex fluorescent detection reagents v2 (Advanced Cell Diagnostics, catalog number: 323110)

i. RNAscope^®^ Multiplex FL v2 AMP 1

ii. RNAscope^®^ Multiplex FL v2 AMP 2

iii. RNAscope^®^ Multiplex FL v2 AMP 3

iv. RNAscope^®^ Multiplex FL v2 HRP-C1

v. RNAscope^®^ Multiplex FL v2 HRP-C2

vi. RNAscope^®^ Multiplex FL v2 HRP blocker

vii. RNAscope^®^ Multiplex FL v2 DAPI

c. RNAscope^®^ Scope Multiplex TSA buffer (Advanced Cell Diagnostics, catalog number: 322809)

d. RNAscope^®^ 50× wash buffer (Advanced Cell Diagnostics, catalog number: 310091)

3. Tyramide signal amplification (TSA) Plus fluorophores

a. TSA Vivid^TM^ fluorophore 570 (Advanced Cell Diagnostics, catalog number: 323272)

b. TSA Vivid^TM^ fluorophore 650 (Advanced Cell Diagnostics, catalog number: 323273)

4. Primary antibodies

a. Mouse anti-human 271 (R&D Systems, catalog number: MAB367, RRID: AB_2152546)

b. Rabbit anti human CD45 (Sigma-Aldrich, catalog number: HPA000440, RRID: AB_611377)

5. Secondary antibodies

a. AlexaFluor^®^ 488 goat anti-rabbit IgG1 (Jackson ImmunoResearch Labs, catalog number: 111-545-003, RRID: AB_2338046)

b. AlexaFluor^®^ 700 goat anti-rabbit IgG (Thermo Fisher Scientific, catalog number: A-21038, RRID: AB_2535709)

6. HyClone^TM^ Dulbecco’s phosphate buffered saline (DPBS, Cytiva, catalog number: SH30028.02)

7. Tween 20 (Sigma-Aldrich, catalog number: P1379)

8. PBS tablets (Gibco^TM^, catalog number: 18912014)

9. Neo-Clear^TM^, xylene substitute (Sigma-Aldrich, catalog number: 1.09843.5000)

10. Absolute ethanol [Apoteket (Swedish pharmacy), catalog number: 910345]

11. Distilled water

12. Sodium azide (Merck, catalog number: 71289)

13. ProLong Gold antifade mountant (Thermo Fisher Scientific, catalog number: P36930)

14. Cover glass 24 mm × 50 mm (Thermo Fisher Scientific, catalog number: 12-545-F)

15. Carboy >3 L (any supplier)

16. Eppendorf tubes, 0.5, 1.5, and 2 mL (any supplier)

17. Serological pipettes, 5, 10, and 25 mL (any supplier)

18. Paper towel or absorbent paper

19. Normal goat serum (Jackson ImmunoResearch Labs, catalog number: 005-000-121, RRID: AB_2336990)

20. Sodium citrate tribasic dihydrate (Sigma-Aldrich, catalog number: S4641-25G)

21. Sodium chloride (Thermo Fisher Scientific, catalog number: 447302500)

**Solutions**

1. RNAscope^®^ wash buffer 1× (see Recipes)

2. Saline sodium citrate (SSC) (see Recipes)

3. Blocking buffer (see Recipes)

4. Primary antibody dilution buffer (see Recipes)

5. Secondary antibody dilution buffer (see Recipes)

6. IF wash buffer (see Recipes)

**Recipes**

**1. RNAscope^®^ wash buffer 1×**

**Table:** 

Reagent	Concentration	Volume
RNAscope^®^ wash buffer	50×	120 mL
Distilled water	n/a	5.88 L

Warm RNAscope 50× wash buffer up to 40 °C for 10–20 min before preparation of RNAscope^®^ wash buffer 1×. 1× wash buffer may be prepared ahead of time and stored at room temperature for up to one month.

**2. Saline sodium citrate (SSC)**

**Table:** 

Reagent	Quantity
NaCl	175.3 g
Sodium citrate	88.2 g
Distilled water	1 L

*Note: Dissolve the NaCl and sodium citrate in 800 mL of distilled water, then adjust the pH to 7.0 with HCl and adjust the volume to 1 L with additional H_2_O. Sterilize by autoclaving.*

**3. Blocking buffer**

**Table:** 

Reagent	Concentration	Volume
PBS	1×	10 mL
Normal goat serum		1 mL
Sodium azide		100 μL

**4. Primary antibody dilution buffer**

**Table:** 

Reagent	Concentration	Quantity
PBS	1×	10 mL
Normal goat serum		500 μL
Sodium azide		100 μL

**5. Secondary antibody dilution buffer**

**Table:** 

Reagent	Concentration	Quantity
PBS	1×	10 mL
Normal goat serum		200 μL
Sodium azide		100 μL

**6. IF wash buffer**

**Table:** 

Reagent	Concentration	Quantity
PBS	1×	2 tablets
Distilled water		1 L
Tween 20		500 μL

## Equipment

1. HybEZ^TM^ Hybridization System

a. HybEZ^TM^ II Hybridization System oven 110V (Advanced Cell Diagnostics, catalog number: 321711)

b. HybEZ^TM^ Humidity Control Tray with lid (Advanced Cell Diagnostics, catalog number: 310012)

c. RNAscope^®^ EZ-Batch^TM^ Slide Rack (Advanced Cell Diagnostics, catalog number: 310017)

d. HybEZ^TM^ Humidifying Paper (Advanced Cell Diagnostics, catalog number: 310015)

2. Olympus VS120-S6-W slide scanner (Olympus)

3. Leica Stellaris 5 Confocal laser-scanning inverted microscope (Leica Microsystems)

4. Steamer (Russell Hobbs)

5. Incubator/water bath (any supplier)

6. Dry oven (any supplier)

7. P1000, P200, P10 pipettors (any supplier)

8. Plastic Coplin jars (any supplier)

## Software and datasets

1. ZEISS Arivis Pro software (ver. 4.0, Carl Zeiss Microscopy Software Center Rostock GmbH, Germany)

2. Prism software version 10.4.2, GraphPad

3. Microsoft Office Excel

## Procedure

*Notes:*

*1. This procedure is adapted from the RNAscope^®^ Multiplex Fluorescent Reagent kit v2 (assay user manual 323100-USM/Rev Date: 12172019).*

*2. We used formalin-fixed paraffin-embedded (FFPE) bone marrow biopsies from the iliac crest bone of patients, obtained from the Division of Pathology (Department of Clinical Sciences, Lund).*

**A. FFPE sample preparation and pretreatment**

1. Bake slides at 60 °C in a dry oven for 1 h.

2. In a chemical hood, fill two Coplin jars with fresh Neo-Clear^TM^ and another two with fresh 100% ethanol.

3. Place slides in Neo-Clear^TM^ for 5 min at room temperature (RT).

4. Remove the slides and place them in the second jar filled with Neo-Clear^TM^. Incubate for 5 min at RT.

5. Remove the slides and place them in a container with 100% ethanol. Incubate for 5 min at RT.

6. Remove the slides and place them in the second container filled with 100% ethanol. Incubate for 2 min at RT.

7. Remove the slides and place them on a sheet of absorbent paper with the section facing up. Dry the slides in a drying oven at 60 °C for 5 min or until they are completely dry.

8. Start the HybEZ^TM^ oven and set the temperature to 40 °C.

9. Place humidifying paper in the HybEZ^TM^ humidity control tray and wet with distilled water.

10. Insert the covered tray into the oven and let it warm up for 30 min at 40 °C before using it.

11. Prepare 50 mL of fresh RNAscope^®^ 1× target retrieval reagent by adding 36 mL of distilled water to 4 mL of RNAscope^®^ 10× target retrieval reagent in a plastic container.

12. Fill up and start the steamer, place a plastic container with distilled water and another container with RNAscope^®^ 1× target retrieval reagent inside, close the lid, and let them reach 98 °C (it takes 30 min).

13. Once the slides are completely dry, take them out of the oven, lay them on the bench, and add 5–8 drops of RNAscope^®^ hydrogen peroxide to cover each section.

14. Incubate for 10 min at RT.

15. Remove the RNAscope^®^ hydrogen peroxide solution by flicking the slides on absorbent paper. Place them into the RNAscope^®^ EZ-Batch^TM^ slide rack with wash tray filled with distilled water.

16. Repeat with fresh distilled water.

17. Once the steamer has reached the desired temperature, transfer the slides into the distilled water container for 10 s.

18. Transfer the slides to the container containing RNAscope^®^ 1× target retrieval reagent and close the steamer with the lid. Incubate for 15 min.

19. Remove the slides from the steamer and wash them with fresh distilled water for 15 s.

20. Transfer to fresh 100% ethanol for 3 min.

21. Dry the slides in the oven at 60 °C for 30 min.

22. Draw a hydrophobic barrier around each section with a wax pen. Let it dry for 5 min.

23. Load the slides onto the RNAscope^®^ EZ-batch slide holder and secure the clamps.

24. Apply 5 drops of RNAscope^®^ Protease Plus to each section. Place the slide holder in the prewarmed HybEZ^TM^ humidity control tray, close the lid, and put the tray back into the oven.

25. Incubate at 40 °C for 30 min.

26. Reapply the protease and incubate for another 10 min in the oven.

27. Remove the slides and place them into the wash tray with distilled water.

28. Repeat with fresh distilled water.

29. Load the slides onto the RNAscope^®^ EZ-Batch^TM^ slide holder and apply 5 drops of RNAscope^®^ Protease Plus to cover each section. Place the slide holder in the prewarmed HybEZ^TM^ humidity control tray, close the lid, and insert the tray back into the oven.

30. Incubate at 40 °C for 30 min.

31. Reapply the protease and incubate in the oven for another 10 min.

32. Remove the slides from the oven and place them into the RNAscope^®^ EZ-Batch^TM^ slide holder with the wash tray filled with distilled water.

33. Repeat with fresh distilled water.

**B. Preparation of materials for hybridization**

1. Prepare RNAscope^®^ wash buffer 1× (see Recipes).

2. Prepare the *TGFB1* and *PDGFA1* RNAscope^®^ probes.

a. Warm probes for 10 min at 40 °C in a water bath.

b. Let probes cool down to RT.

c. Spin the probes down to collect the liquid at the bottom of the tubes.

d. Pipette 1 volume of RNAscope^®^ Multiplex FL v2 HRP-C2 and RNAscope^®^ Multiplex FL v2 HRP-C3 (if applicable) to 50 volumes of RNAscope^®^ Multiplex FL v2 HRP-C1 probe into a new tube.

e. Invert several times to mix.

*Note: Do not mix probes of the same channel. Mixed probes may be stored at 2–8 °C for up to six months.*

3. Equilibrate reagents

a. Remove AMP1, AMP2, AMP3, HRP-C1, HRP-C2, HRP-C3, and HRP blockers from the refrigerator and place at RT.

b. Ensure the HybEZ^TM^ oven and the humidity control tray are at 40 °C.

4. Prepare TSA fluorophores

a. Determine the volume of TSA fluorophores needed (100 μL per sample).

b. Dilute TSA 570 and 650 with TSA buffer to a final concentration of 1:1,500.

**C. Probe hybridization**

1. Remove excess liquid from the slides. Insert the slide holder into the HybEZ^TM^ humidity control tray.

2. Add 4–6 drops of the appropriate probe mix to cover each sample.

3. Close the tray and insert it into the HybEZ^TM^ oven for 2 h at 40 °C.

4. Remove the HybEZ^TM^ humidity control tray from the oven, remove the slide holder, and place the tray back into the oven.

5. Pour 1× wash buffer into the RNAscope^®^ wash tray. Wash the slides for 2 min at RT.

6. Repeat the wash step with fresh buffer.

**Pause point:** Store slides in 5× SSC (see Recipes) overnight at RT. Before resuming the assay, wash twice with 1× wash buffer for 2 min at RT.

**D. Amplification**

*Note: All three amplification steps must be done regardless of the number of probes being used.*

1. Remove excess liquid from the slides and place the slide holder into the HybEZ^TM^ humidity control tray.

2. Add 4–6 drops of RNAscope^®^ Multiplex FL v2 Amp1 to cover each sample.

3. Close the tray and insert the slides into the HybEZ^TM^ oven for 30 min at 40 °C.

4. Remove the HybEZ^TM^ humidity control tray from the oven, remove the slide holder, and place the tray back into the oven.

5. Place the RNAscope^®^ EZ-batch slide holder into the wash tray filled with 1× wash buffer.

6. Wash 3 times for 5 min each with wash buffer.

7. Remove excess liquid from the slides and insert the slide holder into the HybEZ^TM^ humidity control tray.

8. Add 4–6 drops of RNAscope^®^ Multiplex FL v2 to cover the samples.

9. Close the tray and insert it into the HybEZ^TM^ oven for 30 min at 40 °C.

10. Remove the HybEZ^TM^ humidity control tray from the oven. Remove the slide holder and place the tray back into the oven.

11. Pour at least 30 mL of 1× wash buffer into the wash tray.

12. Place the slide holder into the wash tray and wash the slides for 5 min at RT. Repeat two more times.

**E. Development of HRP signal**

1. Remove excess liquid and add 4–6 drops of RNAscope^®^ Multliplex FL v2 HRP-C1.

2. Insert the slide holder into the HybEZ^TM^ humidity control tray, close the tray, and insert it into the HybEZ^TM^ oven for 15 min at 40 °C.

3. Pour at least 30 mL of wash buffer into the wash tray.

4. Remove excess liquid.

5. With the lights off, add 100–150 μL of diluted TSA 570 to each slide.

6. Insert the slide holder into the HybEZ^TM^ humidity control tray, close the tray, and insert it into the HybEZ^TM^ oven for 30 min at 40 °C.

7. Pour at least 30 mL of wash buffer into the wash tray and wash the slides twice for 2 min at RT.

8. Remove excess liquid and add 4–6 drops of RNAscope^®^ Multiplex FL v2 HRP blocker to cover each sample.

9. Insert the slide holder into the HybEZ^TM^ humidity control tray, close the tray, and insert it into the HybEZ^TM^ oven for 15 min at 40 °C.

10. Pour at least 30 mL of 1× wash buffer into the wash tray.

11. Remove the slide holder from the HybEZ^TM^ humidity control, place it in the wash tray, and wash twice for 2 min at RT.

12. Remove excess liquid and add 4–6 drops of RNAscope^®^ Multiplex FL v2 HRP-C2 to cover each sample.

13. Insert the slide holder into the HybEZ^TM^ humidity control tray, close it, and insert it into the HybEZ^TM^ oven for 15 min at 40 °C.

14. Pour at least 30 mL of 1× wash buffer into the wash tray.

15. Remove the slide holder from the HybEZ^TM^ humidity control, place it in the wash tray, and wash twice for 2 min at RT.

16. Remove excess liquid and add 100–150 μL of diluted TSA 650 to each sample.

17. Insert the slide holder into the HybEZ^TM^ humidity control tray, close it, and insert it into the HybEZ^TM^ oven for 30 min at 40 °C.

18. Pour at least 30 mL of 1× wash buffer into the wash tray.

19. Remove the slide holder from the HybEZ^TM^ humidity control, place it in the wash tray, and wash twice for 2 min at RT.

20. Remove excess liquid and add 4–6 drops RNAscope^®^ Multiplex FL v2 HRP blocker.

21. Insert the slide holder into the HybEZ humidity control tray, close it, and insert it into the HybEZ^TM^ humidity control tray. Close it and insert it into the HybEZ^TM^ oven for 15 min at 40 °C.

22. Pour at least 30 mL of 1× wash buffer into the wash tray.

23. Remove the slide holder from the HybEZ^TM^ humidity control, place it in the wash tray, and wash twice for 2 min at RT.

**F. Immunofluorescent staining**

1. Prepare blocking buffer: PBS with 10% normal goat serum and 0.1% sodium azide.

2. Prepare primary antibody dilution buffer: PBS with 5% normal goat serum and 0.1% sodium azide.

3. Block slides for 20 min at RT with blocking buffer in a moisture chamber.

4. Flick slides to remove the liquid and carefully wipe around the sections.

5. Add primary antibodies diluted in primary antibody dilution buffer (mouse anti-human CD271 1:200; rabbit anti-human CD45 1:100).

6. Incubate at 4 °C overnight in a moisture chamber.

7. Place slides in a Coplin jar with wash buffer and wash 3 times for 5 min each.

8. Flick slides to remove excess liquid and wipe around the sections with a wipe.

9. Dilute secondary antibodies in secondary antibody dilution buffer (1:500 for both secondary antibodies).

10. Incubate the sections with the secondary antibody mix for 60 min at RT in the dark in a moisture chamber.

11. Place slides in a Coplin jar with wash buffer and wash slides three times for 5 min each.

12. Remove excess liquid from slides and add ~50 μL of DAPI (dilution 1:1,000) to cover each sample.

13. Incubate for 30 s at RT.

14. Remove DAPI by flicking the slides.

15. Place 1–2 drops of ProLong Gold antifade mountant.

16. Place a 24 mm × 50 mm glass coverslip over the tissue section.

17. Dry slides for 30 min to overnight in the dark.

18. Store slides in the dark at 2–8 °C.

19. Image slides after 8 h and within two weeks. An example is shown in Figure 1.

**Figure 1.**
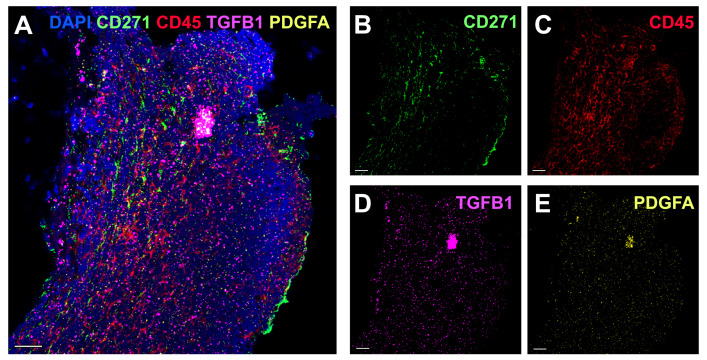
*TGFB1* and *PDGFA1* spatial expression analysis in an acute lymphoblastic leukemia (ALL) sample (fibrosis grade 2). (A) Five-channel overlay of RNAscope and immunofluorescence (IF) antibody staining. Nuclei were stained with DAPI (blue), MSCs with CD271 conjugated to secondary-AF488 in green (B), hematopoietic cells with CD45 conjugated to secondary-AF700 in red (C), RNAscope^®^ probe for TGFB1-C2 coupled to TSA Vivid^TM^ 650 in fuchsia (D), and RNAscope^®^ probe for PDGFA1-C1 coupled to TSA Vivid^TM^ 570 in yellow (E). Scale bar, 25 μm. Scanning of these samples was performed using a 10× objective.

## Data analysis

Arivis’ intensity threshold segmenter was used to generate *TGFB1* and *PDGFA1* objects and to segment nuclei. The surface size of the resulting objects was exported from the Arivis software to Microsoft Excel, in which the expression values were normalized by dividing the total surface of all nuclei objects by the total surface of *TGFB1* or *PDGFA1* objects. To obtain the ratio of *TGFB1* and *PDGFA1* expression in megakaryocyte (MK) vs. non-MK region, the surface size of *TGFB1* and *PDGFA1* objects in MK regions was divided by the corresponding surface in non-MK regions. Arivis Pro (v4.2.1) was used to measure the intensity of *TGFB1* and *PDGFA1* expression in single cells. Single CD271^+^, CD45^+^, and CD45-CD271- cells and megakaryocytes were manually identified, and the sum intensity of *TGFB1* and *PDGFA1* expression signal in each individual cell was determined. The expression levels were compared between different cell types by using the Kruskal–Wallis’s test (Prism software version 10.4.2, GraphPad). Results of data analysis can be found in Bräunig et al. [3].

## Validation of protocol

This protocol has been used and validated in the following research article:

• Bräunig et al. [3]. Combined Multicolor Immunofluorescence Staining and Spatial in Situ messenger RNA Expression Analysis Identifies Potential Fibrosis Drivers in Acute Lymphoblastic Leukemia. *Laboratory Investigation* (Figures 3 and 5)

## General notes and troubleshooting

**General notes**

1. When drawing the hydrophobic barrier, it is essential not to touch the tissue section.

2. The reagents included in the RNAscope Multiplex Fluorescent Reagent kit v2 are stable for nine months after the manufacturing date when stored as indicated by the manufacturer.

3. Do not let samples dry out except when explicitly instructed to do so.

4. TSA vivid dyes dilutions should be prepared freshly for each assay. It is not recommended to store or reuse the TSA working solutions.

5. The hybridization step for the RNA probes is essential, as the sensitivity of the method depends on the appropriate coupling of the probes with the adequate target. Make sure that the samples are covered by the probe mix and the oven is set at the correct temperature.

6. Avoid exposing the samples to light once the fluorophores have been added (from step E5).

7. Antibody concentrations for IF should be optimized for each sample type. Antibody titration is recommended.

8. The use of RNAscope^®^ coupled with antibody-based protein detection for targets other than the ones described in this protocol (transcripts TGFB1 and PDGFA1 and proteins CD45 and CD271) is possible but would probably need optimization depending on the type of tissue, relative abundance of targets, and/or sensitivity to retrieval conditions.

9. Please note that although we have not included the corresponding negative controls in Figure 1, these were performed by (1) using the negative control probes provided in the RNAscope^®^ kit and (2) omitting the primary antibodies to confirm that secondary antibodies did not bind to unspecific targets. We did not observe any unspecific signal in the negative control samples.

**Troubleshooting**

**Problem 1:** Weak fluorescent signal.

Possible cause (1): Tissue has been overfixed.

Solution (1): Increase the target retrieval time up to 1 h.

Possible cause (2): Temperature during hybridization is not stable.

Solution (2): Prewarm the HybEZ^TM^ oven with the HybEZ^TM^ humidity control tray at 40 °C for at least 30 min before using it.

Possible cause (3): Fluorophores/antibodies are too diluted.

Solution (3): Increase concentration of the TSA dyes from 1:1,500 to 1:1,000 or increase antibody concentration.

**Problem 2:** High background signal.

Possible cause (1): Insufficient washing.

Solution (1): Increase the number of washing steps or their duration.

Possible cause (2): Sample endogenous fluorescence or nonspecific binding of RNA probes or secondary antibodies.

Solution (2): Use negative-control RNA probes and include unstained samples to check for autofluorescence.

Possible cause (3): Fluorophores/antibodies are too concentrated.

Solution (3): Increase the dilution of TSA dyes or antibodies.
